# Classification-based comparison of pre-processing methods for interpretation of mass spectrometry generated clinical datasets

**DOI:** 10.1186/1477-5956-7-19

**Published:** 2009-05-14

**Authors:** Wouter Wegdam, Perry D Moerland, Marrije R Buist, Emiel Ver Loren van Themaat, Boris Bleijlevens, Huub CJ Hoefsloot, Chris G de Koster, Johannes MFG Aerts

**Affiliations:** 1 Department of Gynaecologic Oncology, Academic Medical Center, University of Amsterdam, Amsterdam, the Netherlands; 2 Bioinformatics Laboratory, Department of Clinical Epidemiology, Biostatistics and Bioinformatics, Academic Medical Center, University of Amsterdam, Amsterdam, the Netherlands; 3 Clinical Proteomics Group, Department of Medical Biochemistry, Academic Medical Center, University of Amsterdam, Amsterdam, the Netherlands; 4 Swammerdam Institute for Life Sciences, University of Amsterdam, Amsterdam, the Netherlands

## Abstract

**Background:**

Mass spectrometry is increasingly being used to discover proteins or protein profiles associated with disease. Experimental design of mass-spectrometry studies has come under close scrutiny and the importance of strict protocols for sample collection is now understood. However, the question of how best to process the large quantities of data generated is still unanswered. Main challenges for the analysis are the choice of proper pre-processing and classification methods. While these two issues have been investigated in isolation, we propose to use the classification of patient samples as a clinically relevant benchmark for the evaluation of pre-processing methods.

**Results:**

Two in-house generated clinical SELDI-TOF MS datasets are used in this study as an example of high throughput mass-spectrometry data. We perform a systematic comparison of two commonly used pre-processing methods as implemented in Ciphergen ProteinChip Software and in the Cromwell package. With respect to reproducibility, Ciphergen and Cromwell pre-processing are largely comparable. We find that the overlap between peaks detected by either Ciphergen ProteinChip Software or Cromwell is large. This is especially the case for the more stringent peak detection settings. Moreover, similarity of the estimated intensities between matched peaks is high.

We evaluate the pre-processing methods using five different classification methods. Classification is done in a double cross-validation protocol using repeated random sampling to obtain an unbiased estimate of classification accuracy. No pre-processing method significantly outperforms the other for all peak detection settings evaluated.

**Conclusion:**

We use classification of patient samples as a clinically relevant benchmark for the evaluation of pre-processing methods. Both pre-processing methods lead to similar classification results on an ovarian cancer and a Gaucher disease dataset. However, the settings for pre-processing parameters lead to large differences in classification accuracy and are therefore of crucial importance. We advocate the evaluation over a range of parameter settings when comparing pre-processing methods. Our analysis also demonstrates that reliable classification results can be obtained with a combination of strict sample handling and a well-defined classification protocol on clinical samples.

## Background

With the use of mass spectrometry techniques such as MALDI-TOF and SELDI-TOF, it has become possible to analyse complex protein mixtures as found in serum relatively quickly. This has led to the discovery of a large number of proteins and protein profiles associated with various types of diseases [1-4]. However, after promising initial reports important questions have been raised about the reproducibility and reliability of the technique [5]. Reasons for these shortcomings range from pre-analytical effects like sample storage and number of freeze-thaw cycles [6] to the analytical problems of bias due to overfitting and lack of external validation. As a result research moved forward towards the formulation of study requirements and adequate standards in clinical proteomics [7-9]. One of these efforts towards standardization of pre-analytical variables is now being undertaken by the Specimen Collection and Handling Committee of the HUPO Plasma Proteome Project [10].

In this study we investigate some of the problems associated with the generation and analysis of SELDI-TOF MS datasets. In order to eliminate potential pre-analytical biases due to sample handling, we used strict protocols for sample collection, storage and experiments [10].

Pre-processing is the first essential step in the analysis of mass spectrometry generated data. Inadequate pre-processing has been shown to have a negative effect on the reproducibility of biomarker identification and the extraction of clinically useful information [11,12]. Since there is no generally accepted approach to pre-processing, different methods have been proposed, for example [13-17]. Given the large number of existing pre-processing techniques, one would like to know which one is most effective. Therefore, the comparison of pre-processing techniques has recently gained new interest. Cruz-Marcelo *et al*. [18] and Emanuele *et al *[19] compared five and nine, pre-processing methods respectively. However, these studies evaluate the strengths and weaknesses of the different methods on simulated data and quality control datasets. Moreover, the performance of a pre-processing method is only evaluated in terms of reproducibility (coefficient of variation) and sensitivity/specificity of peak detection. While providing important information, our goal in this paper is to compare pre-processing methods in a clinical setting with a relevant and measurable objective. A realistic clinical setting is provided for by in-house ovarian cancer and Gaucher disease profiling datasets and our objective is to maximize classification performance across five different classification methods. We compare the method implemented in Ciphergen ProteinChip Software 3.1 with the mean spectrum technique from the Cromwell package [5] in a classification setting. Ciphergen was included since it is still the most commonly used program by researchers processing their data. Cromwell was included since it showed promising results as a viable alternative to the Ciphergen software [5]. Moreover, these two preprocessing packages were consistently among the top three performers in the recent benchmark studies of Cruz-Marcelo *et al*. [18] and Emanuele *et al *[19] mentioned above. Classification methods have been benchmarked on mass spectrometry data before [20,21], however in general using only one pre-processing method [22]. Recently, Meuleman *et al *[23] showed that the normalization step alone already has a significant influence on classification accuracy. Our analysis extends this to an investigation of the influence of the entire pre-processing pipeline and different parameter settings on classification accuracy.

The past few years, both in the microarray [24] and the proteomics [20] field, the importance of proper classification protocols has been pointed out. Core ingredients of such a protocol are (i) complete separation of training data used for estimating the parameters of a model and test data for estimating the accuracy of the model, (ii) multiple estimates of classification accuracy to be able to assess its variance, and (iii) cross-validation on the training data to determine optimal values for hyperparameters of a model and to select features. In this paper, we propose to implement this by a double cross-validation scheme that provides an almost unbiased estimate of the true error.

Combining all of these protocols, ranging from sample collection via pre-processing to classification, we aimed to develop the optimal strategy for analyzing complex mass spectrometry generated datasets such as SELDI-TOF MS datasets.

## Methods

### Samples

For both the ovarian cancer and Gaucher disease data, serum samples were obtained from participating newly diagnosed patients admitted at the Academic Medical Center (AMC) after informed consent was obtained. The study was performed in agreement with the Helsinki Declaration and approved by the Ethical Committee at the Academic Medical Center, University of Amsterdam.

The group of newly diagnosed non-familial epithelial ovarian carcinoma patients consisted of 14 persons of whom 2 patients had stage I/II invasive epithelial ovarian cancer and 12 patients had stage III/IV epithelial ovarian cancer based on FIGO (Fédération Internationale de Gynécologie Obstétrique) criteria. Among the 14 patients with ovarian carcinoma, 11 were serous, 2 were endometroid and 1 was mucinous. Well-matched samples of 14 patients with benign gynecological disease were also collected at the AMC to be used as controls. This group consisted of patients with a serous cystadenoma, mature cystic teratoma, fibroma of the ovary or patients that underwent an abdominal myomectomy. The two groups were matched for age and body mass index (BMI). Mean ages in the groups were 58 (range 27–71) for the ovarian cancer patients and 59 (range 27–70) for the control group. The mean BMI in the group of ovarian cancer patients was 27.2 (range 17.6–42.1) as compared to 28.7 (range 19.5–53.4) in the control group. Both groups had an equal distribution of pre- and post-menopausal patients.

For the Gaucher dataset, patients with Gaucher disease type I referred to the Academic Medical Center were included. The group consisted of 10 males and 9 females, 15–65 years old. All patients were included before initiation of therapy. The control group consisted of 7 male and 13 female healthy volunteers, 23–68 years old.

Samples from both ovarian cancer and Gaucher patients were collected before treatment using a strict protocol. Blood was collected in the morning at a minimum of two hours after the patient's last meal and left to clot for thirty minutes. After centrifugation (at 1750 × g) serum was immediately frozen and stored at -80°C. Samples used for these experiments were only thawed once.

### SELDI-TOF MS

Plasma samples were analyzed using surface-enhanced laser desorption/ionization (SELDI) time of flight (TOF) mass spectrometry (MS). Samples from ovarian cancer patients and controls were processed on the CM10 ProteinChip array, a weak cation exchanger, and the Q10 ProteinChip array, a strong anion exchanger (Ciphergen Biosystems, Fremont, California, USA). Samples from Gaucher patients and controls were only processed on CM10 ProteinChip arrays. Samples were thawed and centrifuged at 16,000 rpm for 5 minutes. 10 μl of each serum sample was denatured in 90 μl U9 mix (2.2 M Thio/7.7 M urea, 2% CHAPS (3 [(3-cholamidopropyl)dimethylammonio]-propane-sulfonicacid), and 1% dithiothreitol (DTT)) at room temperature for 60 minutes. 10 μl of this solution was mixed with 90 μl binding buffer (50 mM phosphate buffer, pH 6.0, 0.1% Triton X-100) and added to a CM10 ProteinChip array. For the Q10 ProteinChip array 10 μL of this solution was mixed with 90 μL binding buffer (50 mM Tris [tris(hydroxymethyl)aminomethane] HCl, pH 8.0, 0.1% Triton X-100), before being added to the ProteinChip array. Before incubation with serum samples, the ProteinChip arrays were washed twice for 5 minutes with binding buffer [25]. In both studies patient and control samples were randomly allocated on each ProteinChip array to avoid confounding of the effect of interest (patient versus control) with chip effects.

After 45 minutes incubation at room temperature the ProteinChips were washed with binding buffer (2 times, 5 minutes). Next, the CM10 ProteinChip arrays were washed with 50 mM phosphate buffer, pH 6 (2 times, 5 minutes) and the Q10 ProteinChip arrays with Tris buffer pH 8 (2 times, 5 minutes). After the final buffer wash each chip was quickly washed with HPLC-grade water and allowed to dry. 5 μL of matrix (sinapinic acid (10 mg/ml) in 50% acetonitrile and 1% trifluoroacetic acid) was added to each spot on the ProteinChip array twice, allowing the applied matrix solution to dry between applications.

The arrays were read on a PBSII reader (Ciphergen Biosystems, Fremont, California, USA) with laser intensities of 175 (ovarian cancer) and 165 (Gaucher), a detector sensitivity of 6 and a detection size range between 1.5 and 20 kDa (ovarian cancer), 1 and 10 kDa (Gaucher). The spectra were calibrated using the All-in-one Peptide Molecular Weight mix (Ciphergen Biosystems, Fremont, California, USA). Peptides used for calibration were bovine Insulin β-chain (3495 Da), Human Insulin (5807 Da) and Hirudin BHVK (7033 Da). Calibration was performed once before measuring the CM10 ProteinChip arrays in rapid succession. Q10 ProteinChip arrays were calibrated individually by using a spot on every ProteinChip array for calibration mixture.

### Pre-processing and peak detection using ProteinChip Software

Pre-processing was done with the commercial ProteinChip Software (version 3.1.1, Ciphergen Biosystems) and its Biomarker Wizard module. Baseline correction was applied to all spectra. The algorithm is a modified piecewise convex-hull that attempts to find the bottom of the spectra and correct the peak height and area [26]. Spectra were normalized to the average total ion current (TIC) in the mass range from 1.5 to 50 kDa (ovarian cancer), 1 to 10 kDa (Gaucher).

On the Gaucher dataset spot-to-spot calibration was performed. A set of peaks that is present in all our spectra was chosen to determine the correction factors for the different positions on a ProteinChip array. The correction factors were applied to the corresponding mass spectra and used in the recalculation of the masses.

Peak detection was performed with the Biomarker Wizard module in the mass range from 1.5 to 50 kDa (ovarian cancer), 1 to 10 kDa (Gaucher). Biomarker Wizard groups peaks of similar molecular weight across spectra. The algorithm is divided in two passes, the first pass detects well-defined peaks with a high specificity and forms clusters around them. In the second pass smaller peaks are added to an existing cluster. Four parameters have to be specified for peak detection with Biomarker Wizard: (i) First Pass (signal-to-noise ratio (S/N)), (ii) Min Peak Threshold (% of all spectra), (iii) Cluster Mass Window (% of mass), and (iv) Second Pass (S/N). The First Pass S/N threshold specifies the sensitivity of the first pass of peak detection. The Min Peak Threshold is the minimum number of spectra, as a percentage of all spectra, in which a peak must be present in order to form a cluster. A cluster will not be formed around a peak if it is not present in the requisite number of spectra, and the label is deleted. Cluster Mass Window specifies the width of the mass window as a percentage of a peak's mass. This determines the width of a cluster as a function of molecular weight. The Second Pass S/N threshold specifies how to populate the clusters from the first pass with peaks that were too small to be found in the first pass.

For both datasets, we used three typical different peak detection settings with increasing stringency (Ciphergen A-C, Table 1) in Biomarker Wizard to evaluate the effect of peak detection on classification outcome. Setting A was chosen in such a way that many – potentially noisy – peaks were detected. Settings B and C are more stringent by increasing the signal-to-noise ratio of detected peaks or the number of spectra in which a peak must be present. This way we covered a broad range of detected peaks, going from about 100 (setting C) to 500–800 (setting A). Any peak intensity that was zero or negative after baseline correction was set equal to half the minimum of the positive corrected intensities for that peak. Resulting peak intensities were log2-transformed in order to stabilize their variance. In the rest of the paper we will refer to these pre-processing steps as Ciphergen pre-processing.

**Table 1:** Peak detection settings used in Ciphergen pre-processing

	First pass	Threshold	Mass window	Second pass
Ovarian cancer				

Ciphergen A	5	30	0.3	2
Ciphergen B	10	30	0.3	5
Ciphergen C	12	40	0.4	5

Gaucher				

Ciphergen A	2	20	0.3	1
Ciphergen B	3	30	0.3	2
Ciphergen C	5	30	0.3	2

### Pre-processing and peak detection using Cromwell software

Pre-processing was done with the publicly available Cromwell software developed by the bioinformatics group at the MD Anderson Cancer Center [14]. From the raw spectra, a mean spectrum was calculated by averaging all spectra. Smoothing of the mean spectrum was done via wavelet denoising using the undecimated discrete wavelet transform (UDWT). Because most signals can be represented by a small number of wavelet coefficients and white noise is distributed equally among all wavelet coefficients, this approach denoises spectra with minimal attenuation of the features of the signal [5,14]. The UDWT is invariant under linear shift. The wavelet smoothing threshold was set at 10. Baseline correction was performed by computing a monotonic local minimum curve on the denoised signal. Normalization of the mean spectrum was done by dividing by the total ion current within the given mass range. Peaks were identified from this denoised, baseline corrected and normalized mean spectrum as a local maximum with S/N greater than a user-defined threshold (see below) together with the nearest local minima to the left and the right, respectively, of the local maximum. The interval between the two bordering minima of a peak in the average spectrum was used to define peak positions.

Peaks identified from the mean spectrum were quantified in the individual spectra. First, all spectra were denoised, baseline corrected, and normalized. Individual smoothed spectra were searched for the maximum within each peak interval defined on the mean spectrum, taking the corresponding intensity values as individual values for these peaks. Resulting peak intensities were multiplied with a factor 1000 to bring them on a similar scale as Ciphergen data and then log2-transformed.

We adapted the Cromwell MATLAB (The Mathworks Inc, Natick, Massachusetts, USA) scripts in various ways. A script was written to read in the raw data (XML) into an appropriate Matlab structure. Furthermore, in order to fit an accurate baseline, the low-mass area of the spectra that is saturated with matrix peaks was ignored using a conservative cut-off of 1500 Da (ovarian cancer dataset) or 1000 Da (Gaucher dataset). The spectra were also truncated at their high-mass end with a cut-off of 50000 Da (ovarian cancer dataset) or 10000 Da (Gaucher dataset). This was done because a long noisy tail can bias smoothing towards the noise. A further addition to the original Cromwell software as described by Coombes *et al*. [14] was made in the form of spectral alignment. Visual inspection showed that the individual spectra were not well aligned (data not shown). A time warping alignment to correct for horizontal shift was executed on the basis of manually selected peaks using the function "msalign" from the Matlab Bioinformatics toolbox. The peaks used for alignment were m/z values 2750, 5914, 6441 and 6639 Da for CM10 and 4095 and 15946 Da for Q10 (ovarian cancer dataset) and 2735, 3391, 4170, and 9299 (Gaucher dataset).

As with Ciphergen pre-processing, we used three different peak detection settings with increasing stringency for S/N. We used S/N settings of 1, 3, and 5 (Cromwell A-C). This way we covered a broad range of detected peaks, going from about 100 (setting C) to 200–500 (setting A). Note that a signal-to-noise ratio of 3–5 has been recommended by the developers of Cromwell [14].

### Datasets

For the ovarian cancer dataset, CM10 and Q10 ProteinChip array data were analyzed with three different pre-processing settings for both Cromwell and Ciphergen (12 configurations). For each pre-processing method and settings we also made a combined CM10/Q10 dataset by taking the union of the CM10 and Q10 peak intensities for each sample (6 configurations). In total we therefore used 18 configurations for investigation of differential expression and prediction of patient status for cancer versus control. For the Gaucher dataset, CM10 ProteinChip array data was analyzed with three different pre-processing settings for both Cromwell and Ciphergen (6 configurations).

### Statistical Analysis

T-tests were used to identify differentially expressed peaks between patient and control groups. Analysis of variance (ANOVA) was also used to assess the importance of label (patient versus control), chip (array), and interaction effects between label and chip within the dataset. Resulting p-values were corrected for multiple testing using the Benjamini-Hochberg False Discovery Rate (FDR) adjustment [27]. Tests were considered to be significant if the adjusted p-values were < 0.2.

### Classification

To test whether patient status (control/diseased) can be predicted from the peak profiles obtained, five classification methods often used for microarray and proteomics data were compared. The models are classification trees, linear support vector machines (SVM), DLDA (Diagonal Linear Discriminant Analysis), naive Bayes with Gaussian class-conditional densities (Diagonal Quadratic Discriminant Analysis (DQDA)) [28] and PCDA (Principal Component Discriminant Analysis) [29].

The models were validated with repeated random sampling methodology as advocated by Michiels *et al*. [30]. Random splits of each dataset of *N *samples were performed to generate 1000 (ovarian cancer dataset) or 500 (Gaucher dataset) different training sets (size *n*) and 1000 respectively 500 associated test sets (size *N-n*). In each of the random splits, the number of samples for both classes was balanced in both training and test set. The accuracy of the resulting classifier was assessed on the corresponding test set. We report average accuracy on the test sets and its corresponding confidence interval. To investigate the influence of the training set size on the accuracy of the classifier, we also varied the training set size (*n *= 6,8,...,26 for the ovarian cancer dataset, *n *= 9,11,...,37 for Gaucher). Estimation of the optimal values for hyperparameters of the models was done with 5-fold cross-validation on the training set. Such a double cross-validation scheme provides an almost unbiased estimate of the true error [31]. Hyperparameters to be estimated and their possible values were: complexity parameter (classification trees), cost parameter (0.5,1,...,3; SVMs), number of principal components (1–10; PCDA)

All classifiers were used under three different regimes. In the first regime, all peaks are used to estimate the model parameters. The second regime uses feature selection to extract the peaks most informative for predicting patient status. For each training set, an optimal classifier was identified from the 10, 20,...,50 peaks for which expression was most highly correlated with disease as determined by the t-statistics between the two classes. The optimal number of peaks was again selected with 5-fold cross-validation on the training set. In the third regime, class labels were permuted to obtain an estimate of the performance of the classifiers on random data. Statistical comparisons for classifiers over multiple configurations were performed with a non-parametric Friedman test for repeated measures and the Wilcoxon-Nemenyi-McDonald-Thompson post-hoc test [32]. Statistical analyses were performed using Bioconductor packages and in-house scripts in the statistical software package R [33].

## Results

### Peak detection: comparison of Ciphergen and Cromwell

For both Ciphergen and Cromwell pre-processing, three different peak detection settings were chosen. More stringent peak detection parameters indeed resulted in a decrease in the number of peaks selected (Table 2). First, we will illustrate differences in peak detection between Ciphergen and Cromwell with a few specific examples from the ovarian cancer dataset.

**Table 2:** Number of peaks after peak selection

	Ovarian cancer			Gaucher
		
	CM10	Q10	CM10**/**Q10	
Ciphergen A	776	742	1518	579
Ciphergen B	268	182	450	201
Ciphergen C	155	79	234	90
				
Cromwell A	383	226	609	501
Cromwell B	193	113	306	250
Cromwell C	118	70	188	162

Three different settings (A, B, C) were used for both Ciphergen and Cromwell pre-processing.

#### Cromwell detects shoulders

**Figure 1:**
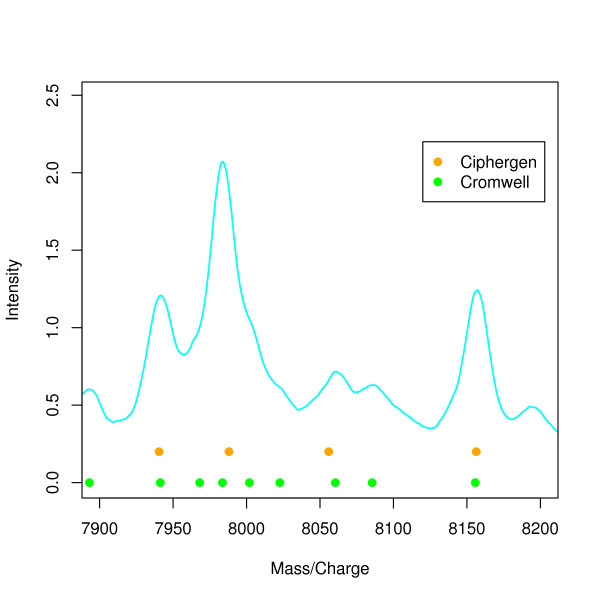
**Comparison of detected peaks (ovarian cancer dataset)**. Plot of the mean (n = 28) raw spectrum illustrating difference in peaks detected by Ciphergen (setting C, orange) and Cromwell (setting C, green) pre- processing. Solid circles below peaks indicate the peak location.

Figure 1 shows an example of peaks detected by either method visualized in a low-intensity region of the mean raw spectrum. Here, all peaks detected by Ciphergen are also detected by Cromwell. In addition, Cromwell detects shoulder peaks near 7968 Da, 8000 Da, and 8023 Da. We consider this to be an advantage of Cromwell since shoulder peaks might very well be biologically relevant and could, for example, be the result of overlapping peaks for peptides with similar mass.

#### Ciphergen detects low intensity peaks

**Figure 2:**
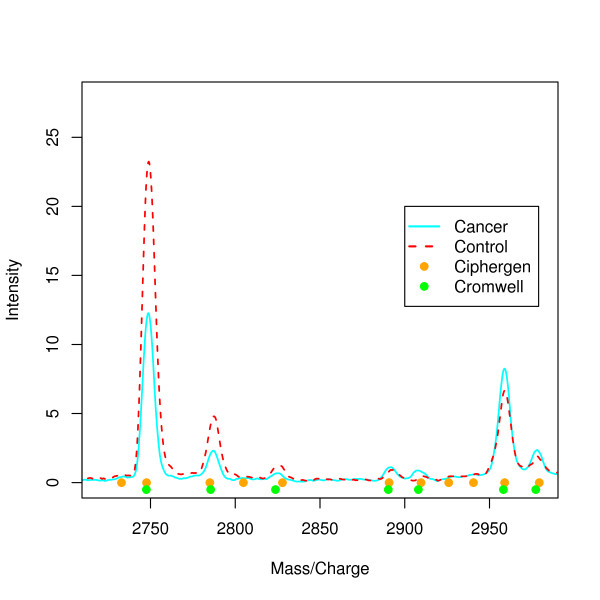
**Comparison of mean raw spectra for cancer and control groups**. Plot of the mean raw spectra calculated for cancer (n = 14, cyan line) and control (n = 14, dashed red line) groups. Solid circles below peaks indicate peaks detected by Ciphergen (setting B, orange) and Cromwell (setting B, green) pre-processing.

Figure 2 illustrates that Ciphergen can detect peaks not found by Cromwell. However, the low-intensity peak at 2733 Da, for example, is probably a spurious peak and this was confirmed by visual inspection of the individual spectra.

#### Cromwell detects peaks present in few samples

**Figure 3:**
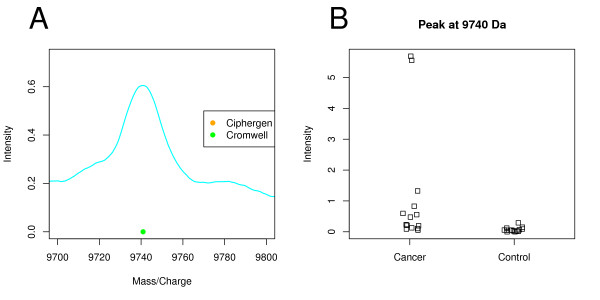
**Peak detected by Cromwell at 9740 Da (ovarian cancer dataset)**. A: plot of the mean raw spectrum of a peak detected by Cromwell (setting C) and not by Ciphergen (setting C). B: dot plot of the 9740 Da peak per sample group (Cromwell, setting C).

As Morris *et al*. [14] observed, Cromwell can still detect peaks if they are only present in part of the spectra. Figure 3A gives an example of such a peak at 9740 Da that goes undetected by Ciphergen. Inspection of the per spectrum intensities of this peak shows that the main contribution to the average spectrum comes from two peaks of moderate intensity in the cancer group (Figure 3B). While such peaks might be biologically interesting, we consider them of less relevance as a potential biomarker because of their large inter-individual variation.

Not withstanding these specific differences, generally the overlap between the peaks detected by either Ciphergen or Cromwell is large. We matched peaks *x *(Ciphergen) and *y *(Cromwell) if | *x *- *y *| < max(0.004*x*, 0.004*y*), that is, if the mass difference between the two peaks is less than 0.4% of the mass of both peaks. The factor 0.004 was chosen since it corresponds to the setting used for the 'Cluster Mass Window' parameter in Ciphergen pre-processing (Table 1). In this way, 105 out of 118 CM10 peaks detected by Cromwell C could be matched to peaks detected by Ciphergen C (ovarian cancer dataset). For the Gaucher dataset, 82 out of 90 peaks detected by Ciphergen C could be matched to peaks detected by Cromwell C. To assess the similarity of the peak intensities, the Pearson correlation coefficient across all samples was calculated for each matched peak pair. Similarity was high: for CM10 (setting C, ovarian cancer dataset) the median correlation for the 105 matched peak pairs was 0.89 (range 0.27 – 0.97). For the Gaucher dataset, the median correlation for the 82 matched peak pairs was 0.91 (range 0.0 – 0.98). Similarity decreases when less strict peak detection settings are used, *e.g*., for CM10 (setting A, ovarian cancer dataset) the median correlation for the matched peaks is 0.63 (range -0.23 – 0.97).

Reproducibility of the pre-processed spectra was addressed by calculating the coefficient of variation (CV) over all peaks and all spectra (see Additional File 1 and Additional File 2). The reproducibility is largely comparable across pre-processing methods. There is a tendency of Ciphergen pre-processing being less variable than Cromwell on the ovarian cancer dataset, especially for the more stringent parameter settings (p < 0.01 for both CM10 and Q10 (setting C)). An opposite tendency was observed on the Gaucher dataset with Cromwell pre-processing being less variable than Ciphergen for the least stringent parameter settings (p < 0.01, setting A).

### Differential expression: comparison of Ciphergen and Cromwell

The correct identification of differentially expressed peaks (corresponding to peptides or proteins) is highly relevant for clinical datasets. Since both our datasets consist of two biologically very distinct classes, we expect a good pre-processing method to identify many peaks with a small FDR when comparing patients and controls. Therefore, an ANOVA was performed to assess the importance of label (patient versus control), chip (array), and interaction effects between label and chip. Both pre-processing methods led to a considerable number of peaks that are differentially expressed between the patient and the control group even when correcting for multiple testing (Table 3, label effect). For Ciphergen pre-processing of the ovarian cancer dataset, there was clear evidence of a chip effect for a number of peaks. The effect was most pronounced for the Q10 dataset (setting A): 57 peaks against 6 in the CM10 dataset (Table 3, chip effect). A likely explanation of the chip effect is the fact that Q10 arrays were calibrated individually with respect to a calibration spot on the array. Nevertheless, no significant interaction effect between patient group and array was found (Table 3, label:chip effect) and none of the peaks that could differentiate between the arrays could differentiate between cancer and control. The chip effect is almost absent in Q10 Cromwell processed data. This provides compelling evidence that our spectral alignment extension of Cromwell can correct for the misalignment caused by per-array calibration. Chip effects are also present in the Gaucher dataset, irrespective of the pre-processing method used. However, these effects are caused by just one Gaucher patient sample and removing the sample from the dataset removes chip effects (data not shown). Since there was almost no interaction effect between patient group and array, we decided to leave this sample in.

**Table 3:** Differential expression

		Ovarian cancer			Gaucher
		
		CM10	Q10	CM10**/**Q10	
label	Ciphergen A	43	19	57	57
	Ciphergen B	37	15	47	46
	Ciphergen C	41	13	50	30

chip	Ciphergen A	6	57	69	8
	Ciphergen B	10	35	52	7
	Ciphergen C	0	15	16	10

label:chip	Ciphergen A	0	0	0	3
	Ciphergen B	0	0	0	0
	Ciphergen C	0	0	0	0

label	Cromwell A	153	17	142	43
	Cromwell B	112	12	95	40
	Cromwell C	67	8	61	29

chip	Cromwell A	0	0	0	22
	Cromwell B	0	0	0	37
	Cromwell C	0	0	0	26

label:chip	Cromwell A	0	0	0	1
	Cromwell B	0	0	0	0
	Cromwell C	0	0	0	0

Number of peaks with an adjusted p-value < 0,2 (ANOVA and correction for multiple testing) for different peak detection settings (A-C for both Ciphergen and Cromwell) for CM10, Q10, and CM10/Q10 (ovarian cancer dataset) and the Gaucher dataset. An ANOVA was performed for label effect (patient versus control), chip (array) effect and interaction effect label:chip.

**Figure 4:**
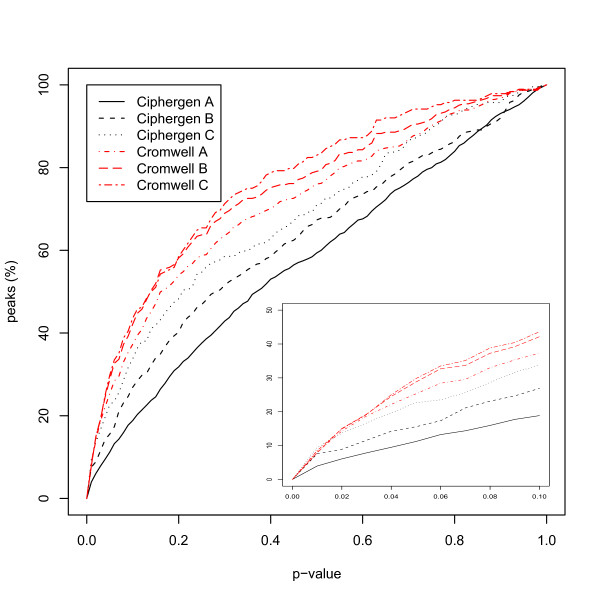
**Cumulative plot of significance of detected peaks (ovarian cancer dataset)**. For each combination of pre-processing method and peak selection settings, the cumulative percentage of peaks with a p-value smaller than the value on the x-axis is shown. P-value of a peak is based on a t-test between the normalized intensities of the cancer and the control group. Inset zooms in on p-values smaller than 0.1. Dataset shown is the combined CM10/Q10 data.

The ANOVA results in Table 3 seem to indicate that for the ovarian cancer dataset – at least for CM10 and CM10/Q10 – Cromwell detects more differentially expressed peaks. Since the total number of peaks detected by Cromwell and Ciphergen using matched settings is smaller (Table 2), this could have been mainly caused by a different multiplicity correction. Therefore, the results in terms of unadjusted p-values are given in Figure 4 (see also Additional Files 3, Additional File 4 and Additional File 5 for other chip types and the Gaucher disease dataset). This confirms the observed trend, although differences are small especially in the range of p-values smaller than 0.01. The overlap between the most differentially expressed peaks detected by either Ciphergen or Cromwell is again large (Table 4). Moreover, the fold changes estimated by either method agree well.

**Table 4:** Comparison of detected peaks (ovarian cancer dataset)

CM10								
Peak (Ciphergen)	logFC	p-value	adjusted p	Peak (Cromwell)	logFC	p-value	adjusted p	cor

1546.06	-0.55	0.004	0.065	1540.24	-0.47	0.001	0.039	0.73
1555.67	-0.83	0.001	0.039	1552.28	-0.39	0.011	0.093	0.77
1584.26	-0.52	0.001	0.034	1540.24	-0.47	0.001	0.039	0.87
2648.88	-0.73	0.001	0.029	1540.24	-0.47	0.001	0.039	0.63
2747.67	-1.28	<0.001	0.008	2747.56	-1.06	0.001	0.039	0.95
2785.04	-1.21	<0.001	0.008	2785.52	-1.08	0.003	0.051	0.95
2824.21	-0.73	0.002	0.044	2823.75	-0.62	0.018	0.105	0.86
3164.30	-0.84	0.001	0.025	3163.52	-0.64	0.013	0.098	0.83
3403.11	-0.81	0.002	0.039	3402.37	-0.68	0.014	0.099	0.93
3441.36	-0.75	0.001	0.031	1615.29	-0.37	0.011	0.093	0.77

Q10								

Peak (Ciphergen)	logFC	p-value	adjusted p	Peak (Cromwell)	logFC	p-value	adjusted p	cor

1721.35	-0.68	0.004	0.046	1511.76	-0.34	0.175	0.439	0.31
4078.66	-0.42	0.015	0.125	4079.56	-0.34	0.155	0.439	0.72
4273.34	-0.48	0.012	0.113	4274.66	-0.28	0.236	0.439	0.75
4287.14	-1.06	<0.001	0.005	4287.39	-0.92	0.009	0.143	0.88
6889.71	-1.02	0.001	0.014	6889.77	-1.43	0.004	0.103	0.92
8574.28	-1.47	<0.001	0.010	8572.2	-1.51	0.004	0.103	0.96
8703.04	-1.05	0.006	0.063	8700.12	-0.96	0.020	0.143	0.95
8772.14	-1.26	<0.001	0.001	8776.73	-1.35	0.004	0.103	0.91
13782.2	-1.11	0.002	0.029	6889.77	-1.43	0.004	0.103	0.91
28143.3	-0.77	0.016	0.125	8682.97	-1.09	0.015	0.143	0.75

List of ten most differentially expressed m/z values after pre-processing with either Ciphergen or Cromwell (setting C) for CM10 and Q10 ProteinChip arrays. For each of the m/z values the log_2 _fold change (logFC), p-value and adjusted p-value are given. Peaks were matched between Ciphergen and Cromwell by calculating Pearson's correlation between a Ciphergen peak profile and all Cromwell peak profiles. The Cromwell peak with highest Pearson's correlation is considered to be a matching peak. Last column gives the corresponding correlation value.

A notable difference between the two methods is the way in which the peak intensities are estimated. Ciphergen takes a two-pass approach to find peaks. If a peak is found in a minimum number of spectra (as determined by the Min Peak Threshold parameter) within a certain mass window, intensities are assigned to spectra without a peak by taking their intensity at the average m/z value of the cluster. In general, this extrapolation will not correspond to a peak in a spectrum. Looking at the peaks with small adjusted p-values as given by the ANOVA (Table 4), the proportion of estimated intensities for a cluster can be as high as ~50%. This is, for example, the case for the 28143Da peak detected on Q10 (Ciphergen C, ovarian cancer dataset). Cromwell does not rely on extrapolated intensities since it locates a local maximum in each pre-processed spectrum. As a consequence, the Ciphergen software assigns different intensities for one and the same m/z value with different peak detection settings, whereas the Cromwell package always assigns the same intensity independent of the peak detection settings. This might explain some of the observed differences.

### Classification: Ciphergen pre-processed data

Classification was done using five different classification methods on both datasets and all configurations. For clarity, results for only one specific setting are summarized in Tables 5 and 6, they are however representative for the overall results (see Additional File 6 and Additional File 7). Classification of the Ciphergen pre-processed datasets was better than chance in the majority of cases (Tables 5 and 6, confidence intervals not including 50%). For the ovarian cancer dataset, the best classification result was obtained on the combined CM10/Q10 data using DLDA or SVM with a mean accuracy on 1000 randomly sampled test sets of 77% (CI: 57–93). There is a large difference in average accuracy depending on the model used, with DLDA giving an 8% higher accuracy than classification trees on the CM10/Q10 data. Combining CM10 and Q10 datasets into a CM10/Q10 dataset is beneficial and increases the mean classification accuracy with on average 4% when compared to the maximum of the results on the individual datasets (Table 5). Classification on CM10 data outperforms Q10 data which is in agreement with the lower number of differentially expressed peaks detected for Q10 (Table 3). For the Gaucher dataset, the best classification result was obtained using SVM with a mean accuracy on 500 randomly sampled test sets of 90% (CI: 75–100). Again there is a large difference in average accuracy between the various models with naive Bayes giving a 17% lower accuracy than SVM.

**Table 5:** Comparison of classifiers (ovarian cancer dataset)

	Ciphergen C			Cromwell A		
	
	CM10**/**Q10	CM10	Q10	CM10**/**Q10	CM10	Q10
DLDA	77 (57–93)	71 (50–93)	71 (50–86)	78 (64–93)	74 (57–93)	70 (43–93)
Naive Bayes	70 (50–93)	66 (43–86)	63 (43–86)	69 (50–86)	68 (50–86)	60 (36–86)
PCDA	73 (50–93)	70 (50–93)	67 (43–86)	77 (57–93)	73 (57–86)	77 (50–93)
SVM	77 (57–93)	71 (50–86)	70 (50–86)	79 (64–93)	77 (57–93)	79 (50–93)
Tree	69 (43–93)	69 (43–93)	64 (36–86)	58 (36–79)	58 (36–79)	52 (29–71)

Average classification accuracy (95% confidence interval) of ovarian cancer versus control on 1000 test sets (size of training sets: 14, size of test sets: 14). Pre-processing was done using setting C for Ciphergen and setting A for Cromwell. Classifiers without feature selection.

**Table 6:** Comparison of classifiers (Gaucher dataset)

	Ciphergen C	Cromwell B
DLDA	77 (50–100)	73 (50–92)
Naive Bayes	73 (50–92)	68 (42–92)
PCDA	81 (58–100)	81 (50–100)
SVM	90 (75–100)	86 (67–100)
Tree	75 (42–92)	72 (42–92)

Average classification accuracy (95% confidence interval) of Gaucher versus control on 500 test sets (size of training sets: 27, size of test sets: 12). Pre-processing was done using setting C for Ciphergen and setting B for Cromwell. Classifiers without feature selection.

**Figure 5:**
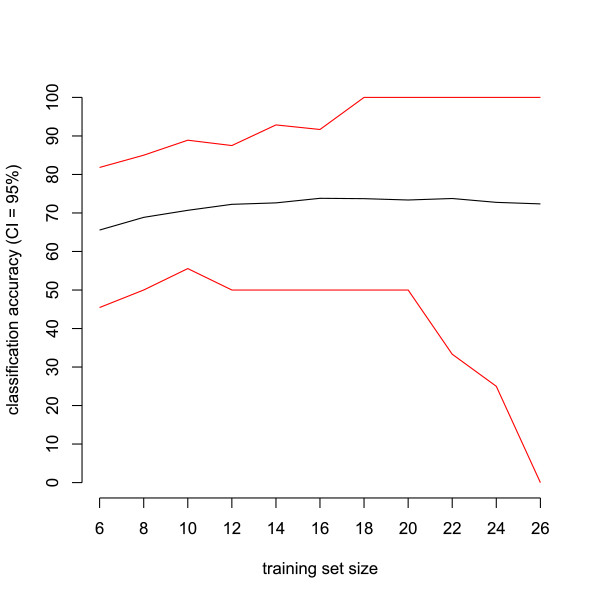
**Classification accuracy using repeated random resampling as a function of corresponding training set sizes**. Average classification accuracy (black line) of ovarian cancer versus control with its 95% confidence interval (red lines) on 1000 test sets as a function of training set size. Dataset used is the CM10 data with Ciphergen pre-processing (setting C) with a DLDA classifier and feature selection.

We also varied the training set size to investigate its influence on the accuracy of the classifier. Figure 5 shows the mean accuracy and its 95% CI as a function of the training set size. Here and for all other cases (data not shown), classification accuracy first increased with a larger training set size and then stabilized.

Different peak detection settings using Ciphergen pre-processing had a considerable influence on classification accuracy. For the ovarian cancer dataset more stringent settings for peak detection, resulting in fewer detected peaks, gave better overall accuracy. Use of feature selection also often led to better classification results in all of the models used apart from SVMs (see Additional File 6). For the Gaucher dataset, there is an opposite tendency of less stringent settings giving better overall accuracy (see Additional File 7).

**Figure 6:**
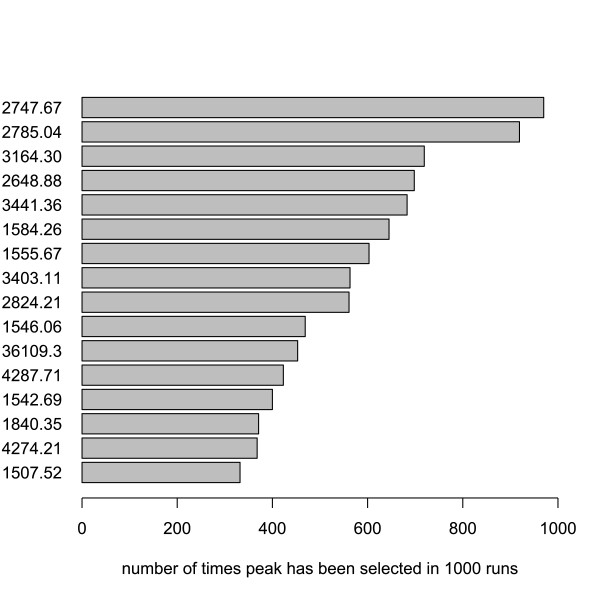
**Histogram of frequently selected peaks**. Peaks (m/z value) selected at least 300 times in 1000 signatures in classification of ovarian cancer versus control. Dataset used is the CM10 data with Ciphergen pre-processing (setting C) with a DLDA classifier and feature selection.

Peak signatures for each of the random splits varied greatly. A typical example of the individual m/z values selected is given in Figure 6: only 16 peaks were included in at least 300 of the 1000 signatures for the ovarian cancer data set. This list gives us candidate proteins with the highest classification potential between ovarian cancer and control patients. The most frequently selected peak at 2748 Da is clearly downregulated in the cancer samples (Figure 2).

### Classification: Cromwell pre-processed data

Classification of Cromwell pre-processed datasets gave similar classification results as for Ciphergen (see Additional Files 6 and Additional File 7). Classification of the Cromwell pre-processed datasets was better than chance in the majority of cases. SVM, PCDA, and to a lesser (on the Gaucher dataset) degree, DLDA again resulted in a higher mean accuracy than naive Bayes and classification trees. For the ovarian cancer dataset, combining CM10 and Q10 data led to increased mean accuracy. Classification on CM10 data often outperforms Q10 data, in agreement with the lower number of differentially expressed peaks detected for Q10 (Table 3). We observed a decrease in classification accuracy on both datasets when more stringent pre-processing parameters were chosen. Use of feature selection during classification did not show a clear trend in terms of classification accuracy.

### Classification: comparison of Ciphergen and Cromwell

**Figure 7:**
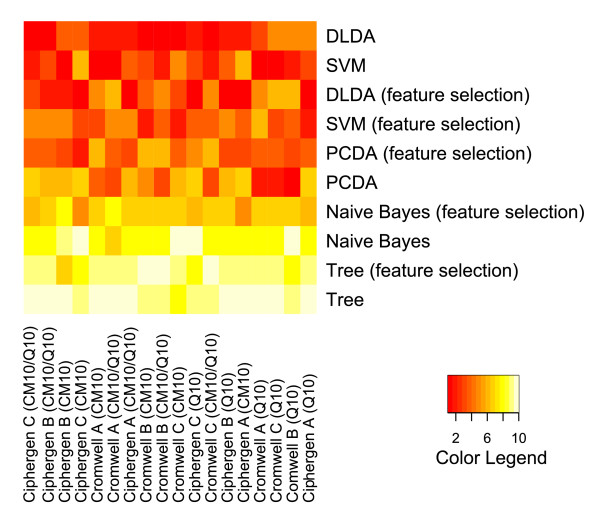
**Comparison of classifiers and pre-processing methods (ovarian cancer dataset)**. Classifiers were ranked by their average classification accuracy on 1000 test sets (size of training sets: 14, size of test sets: 14) for each specific combination of chip type, pre-processing method and peak selection settings. The heatmap gives a colour coding of the ranks from 1 (highest accuracy, red) to 10 (lowest accuracy, light yellow). Classifiers are ordered by their average rank over all combinations, with DLDA being the best ranked classifier. The columns of the heatmap are ranked by their average rank over all classifiers, with Ciphergen pre-processing using setting C and the combined CM10/Q10 data getting the highest rank (see also Additional File 8).

**Figure 8:**
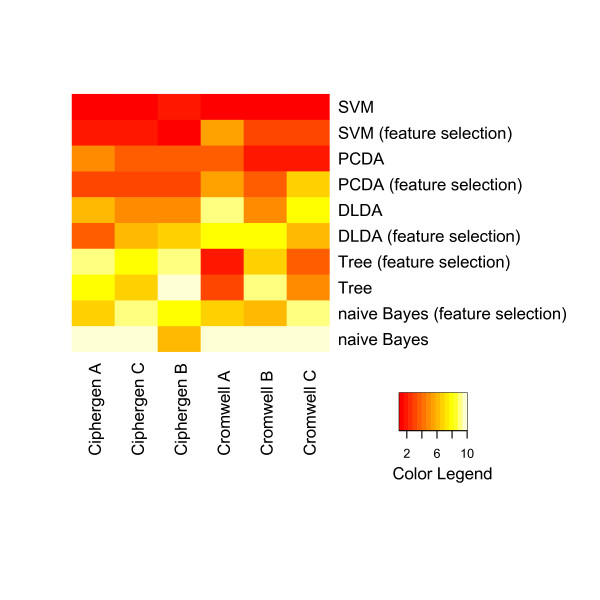
**Comparison of classifiers and pre-processing methods (Gaucher dataset)**. Classifiers were ranked by their average classification accuracy on 500 test sets (size of training sets: 27, size of test sets: 12) for each specific combination of pre-processing method and peak selection settings. The heatmap gives a colour coding of the ranks from 1 (highest accuracy, red) to 10 (lowest accuracy, light yellow). Classifiers are ordered by their average rank over all combinations, with SVM being the best ranked classifier. The columns of the heatmap are ranked by their average rank over all classifiers, with Ciphergen pre-processing using setting A getting the highest rank (see also Additional File 9).

To compare classifiers and pre-processing methods across different datasets, they were ranked based on their average accuracy. The outcome is visualized by heatmaps (Figure 7 and Figure 8, Additional File 8 and Additional File 9). These heatmaps clearly illustrate the trends observed above for each pre-processing method and dataset separately. Differences in methodology between the pre-processing methods are not reflected in a significant difference in classification accuracy. For both pre-processing methods and datasets, PCDA, SVM, and to a lesser degree DLDA, perform significantly better than naive Bayes and classification trees. Also, results on CM10 and combined CM10/Q10 data are significantly better than on Q10 data (ovarian cancer dataset).

## Discussion

In the two clinical studies described here, we have tried to overcome some pre-analytical factors that influence the protein profile in serum unrelated to disease. Careful patient selection, matching for different biological variables and protocolized sample processing has led to datasets that have fewer variables that could bias the classification outcome between patients and controls. Next to issues concerning pre-analytical and analytical factors involved in proteomics, further challenges of mass spectrometry are the pre-processing of spectra and statistical analysis of the detected m/z peaks in relatively small sample sets. To reliably classify such datasets, sound bioinformatics methods are needed that account for variation arising from the biological samples as well as technical variation introduced by sample handling and processing.

Previous comparisons of pre-processing methods were based on the use of tightly controlled calibration (or spike-in) data, quality control data [18], or simulated data [18,19]. Such datasets are highly relevant but capture only part of the complexity observed in clinical samples typically profiled on a mass spectrometer. Moreover, recent benchmark studies focused on comparing pre-processing methods with respect to reproducibility and peak detection. While these are important criteria, it is clear that they do not capture all objectives a good pre-processing method should satisfy. For example, it is easy to minimize the coefficient of variation by eliminating differences between peak intensities across samples even if differences are biologically real. Therefore, we compared two pre-processing methods in a classification setting using five methods on two in-house generated clinical SELDI-TOF MS datasets.

Our comparison of pre-processing methods consisted of the commercial ProteinChip Software of Ciphergen and the mean spectrum approach of Cromwell, a set of publicly available Matlab scripts. While these and other pre-processing methods described in the literature consist of the same basic ingredients (smoothing, baseline subtraction, normalization, peak detection, peak clustering, and peak quantification), the combination of these steps is very different between Ciphergen and Cromwell (see description in Patients, Materials, and Methods). Despite these differences our results indicate that with respect to reproducibility, Ciphergen and Cromwell pre-processing are largely comparable. A recent comparison of various pre-processing algorithms including Ciphergen and Cromwell on quality control data also concluded that, at least for these two pre-processing methods, the difference in reproducibility is small [18]. A comparison of Ciphergen and Cromwell's direct precursor (SUDWT) [5] on quality control data claimed that the reproducibility of Ciphergen pre-processing was significantly lower. However, with their default Ciphergen parameter settings a peak only had to occur in 15% ('Min Peak Threshold') of the spectra to form a peak cluster. Given that Ciphergen determines intensities for missing peaks by extrapolation, a low reproducibility is a direct consequence of such a low threshold. Since our goal is the identification of (a combination of) reliable biomarkers that can discriminate diseased and controls, in this study a peak had to occur in at least 30–40% of the spectra for almost all pre-processing settings (Table 1).

Regarding peak detection we found that the overlap between peaks detected by either Ciphergen or Cromwell is large. This was especially the case for the more stringent peak detection settings. Moreover, similarity of the estimated intensities between matched peaks was high. Also the overlap between the most differentially expressed peaks detected by either Ciphergen or Cromwell is large (Table 4) and estimated fold changes agree well across methods. These results are comparable to those of Cruz-Marcelo *et al*. [18] who found that peak detection with Ciphergen was only slightly more sensitive than with Cromwell for a range of false discovery rates on a simulated data set. The main difference with our comparison is that we used clinical datasets where the 'ground truth', that is the number and location of true peaks, is not known.

As stated above, clinical datasets lack a gold standard that tells us the location of true peaks. However, they in general consist of patient samples of known types or classes. Prediction of patient status therefore offers a highly relevant benchmark for comparison of pre-processing methods on a measurable and objective goal, namely maximization of classification accuracy. We compared five different classification methods and two pre-processing methods on an ovarian cancer and a Gaucher disease dataset generated with two types of ProteinChips. Special care was taken to adequately validate the resulting classifiers. We randomly sampled multiple training and test sets for a range of training set sizes to study the stability of the classifier accuracy. A nested cross-validation procedure was used to simultaneously optimize the number of peaks included in the model and provide an almost unbiased estimate of the true error.

Regarding classification, we conclude that PCDA, SVM, and to a lesser degree DLDA, perform significantly better on all our datasets than naive Bayes and classification trees. A similar observation has been made in a recent comparison of normalization methods for SELDI-TOF MS datasets [23]. In that study SVMs also perform significantly better than classification trees. Moreover, using DLDA, PCDA and SVM almost always led to better than chance classification.

When comparing the classification results from the datasets pre-processed by the two different pre-processing methods, no pre-processing method significantly outperforms the other for all peak detection settings evaluated. However, significant differences are detected within and between pre-processing methods for specific settings. For example, Ciphergen pre-processing with stringent settings (C) on the CM10/Q10 ovarian cancer dataset significantly outperforms Cromwell with stringent settings. Previous comparisons of pre-processing methods were based on one specific parameter setting for each method, see for example [18]. Therefore, they might have detected significant differences caused by a sub-optimal choice of parameter settings for one of the methods compared. Given the large impact of different settings for preprocessing parameters on the overall outcome of classification, evaluating a range of parameter settings should therefore be a routine part of the pre-processing procedure.

In this study, we did not identify the proteins corresponding to the discriminatory peaks, since our focus was on the comparison of different pre-processing and classification methods. Although one does not need to know the protein behind a discriminatory peak for accurate classification, identification of such peaks does give us important additional information. It will help us understand their connection to a specific type of disease and help us discriminate disease-related proteins from artifacts created, for example, during sample preparation.

## Conclusion

We have found that careful patient selection in combination with stringent sampling protocols generates datasets that are suitable for further investigation. Our systematic comparison of two different pre-processing methods using five different classification methods has shown that different pre-processing parameter settings lead to significantly different classification results within and between pre-processing methods. However, for both pre-processing methods, settings could be found that gave comparable maximal classification accuracy on an ovarian cancer and a Gaucher disease dataset. Therefore, we advocate evaluation over a range of different parameter settings when comparing pre-processing methods for mass-spectrometry generated datasets such as SELDI-TOF MS datasets. Also the choice of a suitable classification method is of vital importance for a good classification outcome. Our comparison indicated that PCDA, SVM, and to a lesser degree DLDA, outperform naive Bayes and classification trees, at least on our two datasets.

Comparing different pre-processing methods and parameter settings using maximization of classification accuracy on clinical datasets as prime objective does not only give insight in the quality and reproducibility of the data, but also indicates which pre-processing methods and settings are best suited for a particular dataset. Given the abundance of clinical mass spectrometry generated datasets, a classification-based comparison of pre-processing methods on clinical data is a valuable complement to the use of calibration or simulated data for that purpose.

## List of abbreviations used

ANOVA: analysis of variance; BMI: body mass index; CI: confidence interval; CV: coefficient of variation; DLDA: diagonal linear discriminant analysis; FDR: false discovery rate; FIGO: Fédération Internationale de Gynécologie Obstétrique; HUPO: Human Proteome Organization; MALDI: matrix-assisted laser desorption ionisation; MS: mass spectrometry; PCDA: principal component discriminant analysis; m/z: mass/charge; SD: standard deviation; SELDI: surface enhanced laser desorption ionisation; SN: signal-to-noise; SVM: support vector machines; TIC: total ion current; TOF: time of flight; UDWT: undecimated discrete wavelet transform.

## Competing interests

The authors declare that they have no competing interests.

## Authors' contributions

WW collected samples and clinical information, carried out the SELDI-TOF experiments, interpreted results and drafted the manuscript. PDM carried out the statistical analyses, interpreted the results and drafted the manuscript. MRB was responsible for the design of the study and sample collection. EVLvT contributed to data pre-processing and statistical analyses. BB carried out the SELDI-TOF experiments. HCJH advised on statistical analyses and interpretation of results. CGdK and JMFGA contributed materials and were responsible for the design of the study. All authors read and approved the final manuscript.

## Supplementary Material

Additional file 1 **Variance analysis (ovarian cancer dataset)**. Boxplots of the coefficient of variation (CV, standard deviation/mean peak intensity). Left panel: CV for all combinations of pre-processing method (Ciphergen: cyan, Cromwell: red) and peak selection setting (A, B, C) for the CM10 chip. Right panel: idem for Q10 chip. Click here for file

Additional file 2 **Variance analysis (Gaucher dataset)**. Boxplots of the coefficient of variation (CV, standard deviation/mean peak intensity). CV for all combinations of pre-processing method (Ciphergen: cyan, Cromwell: red) and peak selection setting (A, B, C). Click here for file

Additional file 3 **Cumulative plot of significance of detected peaks (ovarian cancer dataset. CM10)**. For each combination of pre-processing method and peak selection settings, the cumulative percentage of peaks with a p-value smaller than the value on the x-axis are shown. P-value of a peak is based on a t-test between the normalized intensities of the cancer and the control group. Click here for file

Additional file 4 **Cumulative plot of significance of detected peaks (ovarian cancer dataset, Q10)**. For each combination of pre-processing method and peak selection settings, the cumulative percentage of peaks with a p-value smaller than the value on the x-axis is shown. P-value of a peak is based on a t-test between the normalized intensities of the cancer and the control group. Click here for file

Additional file 5 **Cumulative plot of significance of detected peaks (Gaucher dataset)**. For each combination of pre-processing method and peak selection settings, the cumulative percentage of peaks with a p-value smaller than the value on the x-axis is shown. P-value of a peak is based on a t-test between the normalized intensities of the Gaucher and the control group. Click here for file

Additional file 6 **Comparison of classifiers and pre-processing methods (ovarian cancer dataset)**. Average classification accuracy on 1000 test sets (size of training sets: 14, size of test sets: 14) for each specific combination of pre-processing method and peak selection settings. Click here for file

Additional file 7 **Comparison of classifiers and pre-processing methods (Gaucher dataset)**. Average classification accuracy on 500 test sets (size of training sets: 27, size of test sets: 12) for each specific combination of pre-processing method and peak selection settings. Click here for file

Additional file 8 **Comparison of classifiers and pre-processing methods (ovarian cancer dataset)**. Each combination of chip type, pre-processing method and peak selection was ranked by its average classification accuracy on 1,000 test sets (size of training sets: 14, size of test sets: 14) for each classifier. The heatmap gives a colour coding of the ranks from 1 (highest accuracy, red) to 18 (lowest accuracy, light yellow). Columns of the heatmap are ranked by their average rank over all classifiers, with Ciphergen pre-processing using setting C and the combined CM10/Q10 data getting the highest rank. Classifiers are ordered by their average rank over all pre-processing combinations, with DLDA being the best ranked classifier. Click here for file

Additional file 9 **Comparison of classifiers and pre-processing methods (Gaucher dataset)**. Each combination of pre-processing method and peak selection was ranked by its average classification accuracy on 500 test sets (size of training sets: 27, size of test sets: 12) for each classifier. The heatmap gives a colour coding of the ranks from 1 (highest accuracy, red) to 6 (lowest accuracy, light yellow). Columns of the heatmap are ranked by their average rank over all classifiers, with Ciphergen pre-processing using setting A getting the highest rank. Classifiers are ordered by their average rank over all pre-processing combinations, with SVM being the best ranked classifier. Click here for file
