# Supplementary material for: Classification-based comparison of pre-processing methods for interpretation of mass spectrometry generated clinical datasets
Source: Proteome Sci. 2009 May 14;7:19. doi: 10.1186/1477-5956-7-19 (PMC2689848; doi:10.1186/1477-5956-7-19)
Supplement: Additional file 1 — Variance analysis (ovarian cancer dataset). Boxplots of the coefficient of variation (CV, standard deviation/mean peak intensity). Left panel: CV for all combinations of pre-processing method (Ciphergen: cyan, Cromwell: red) and peak selection setting (A, B, C) for the CM10 chip. Right panel: idem for Q10 chip. [file 1477-5956-7-19-S1.pdf]

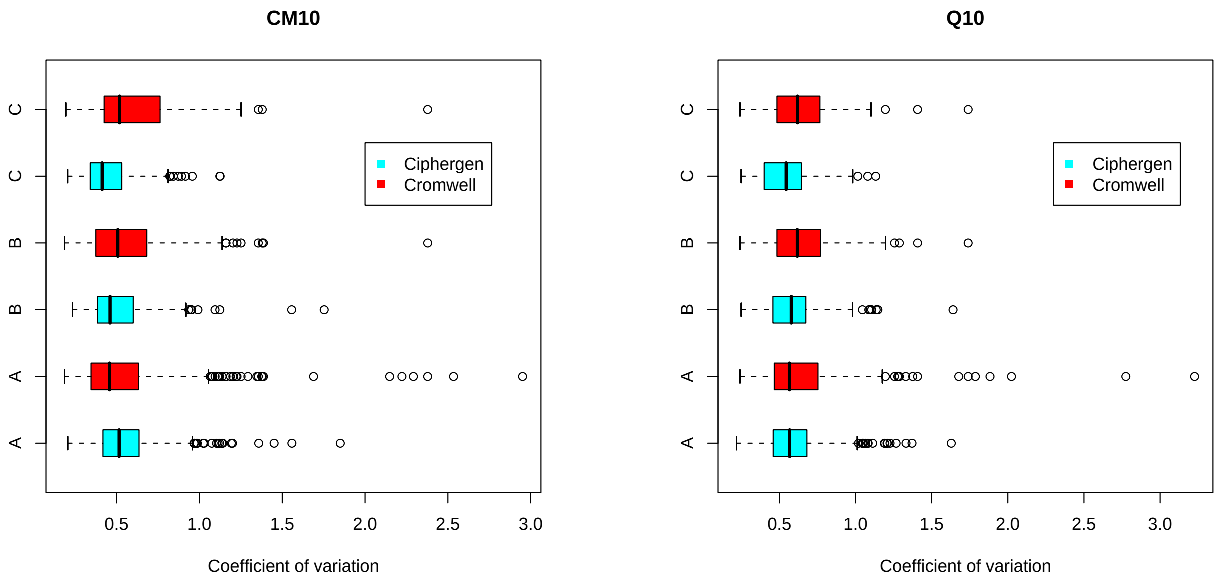

Variance analysis (ovarian cancer dataset). Boxplots of the coefficient of variation (CV, standard deviation/mean peak intensity). Left panel: CV for all combinations of pre-processing method (Ciphergen: cyan, Cromwell: red) and peak selection setting (A, B, C) for the CM10 chip. Right panel: idem for Q10 chip.
