# Supplementary material for: Classification-based comparison of pre-processing methods for interpretation of mass spectrometry generated clinical datasets
Source: Proteome Sci. 2009 May 14;7:19. doi: 10.1186/1477-5956-7-19 (PMC2689848; doi:10.1186/1477-5956-7-19)
Supplement: Additional file 2 — Variance analysis (Gaucher dataset). Boxplots of the coefficient of variation (CV, standard deviation/mean peak intensity). CV for all combinations of pre-processing method (Ciphergen: cyan, Cromwell: red) and peak selection setting (A, B, C). [file 1477-5956-7-19-S2.pdf]

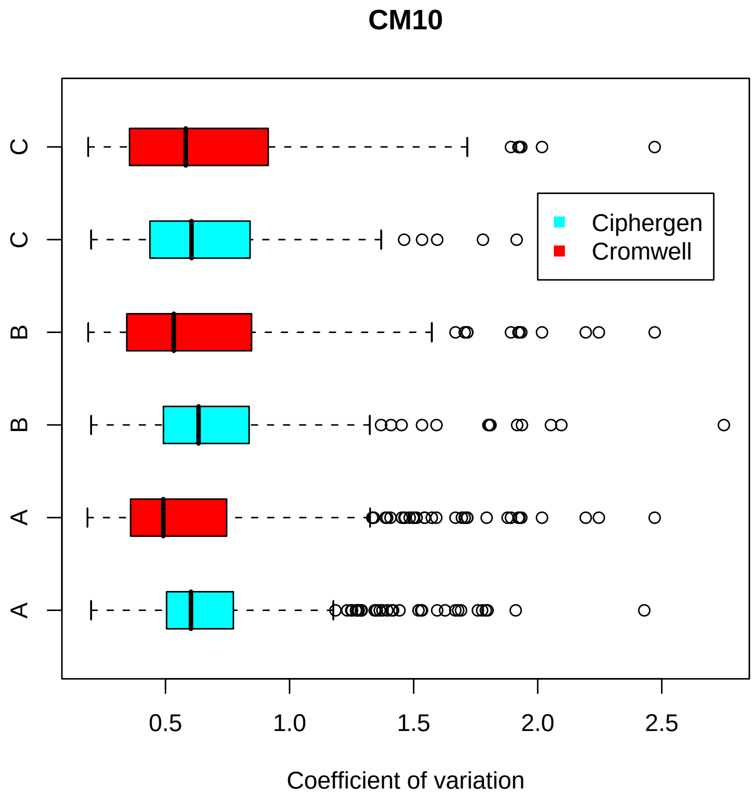

Variance analysis (Gaucher dataset). Boxplots of the coefficient of variation (CV, standard deviation/mean peak intensity). CV for all combinations of pre-processing method (Ciphergen: cyan, Cromwell: red) and peak selection setting (A, B, C).
