# Supplementary material for: Classification-based comparison of pre-processing methods for interpretation of mass spectrometry generated clinical datasets
Source: Proteome Sci. 2009 May 14;7:19. doi: 10.1186/1477-5956-7-19 (PMC2689848; doi:10.1186/1477-5956-7-19)
Supplement: Additional file 3 — Cumulative plot of significance of detected peaks (ovarian cancer dataset. CM10). For each combination of pre-processing method and peak selection settings, the cumulative percentage of peaks with a p-value smaller than the value on the x-axis are shown. P-value of a peak is based on a t-test between the normalized intensities of the cancer and the control group. [file 1477-5956-7-19-S3.pdf]

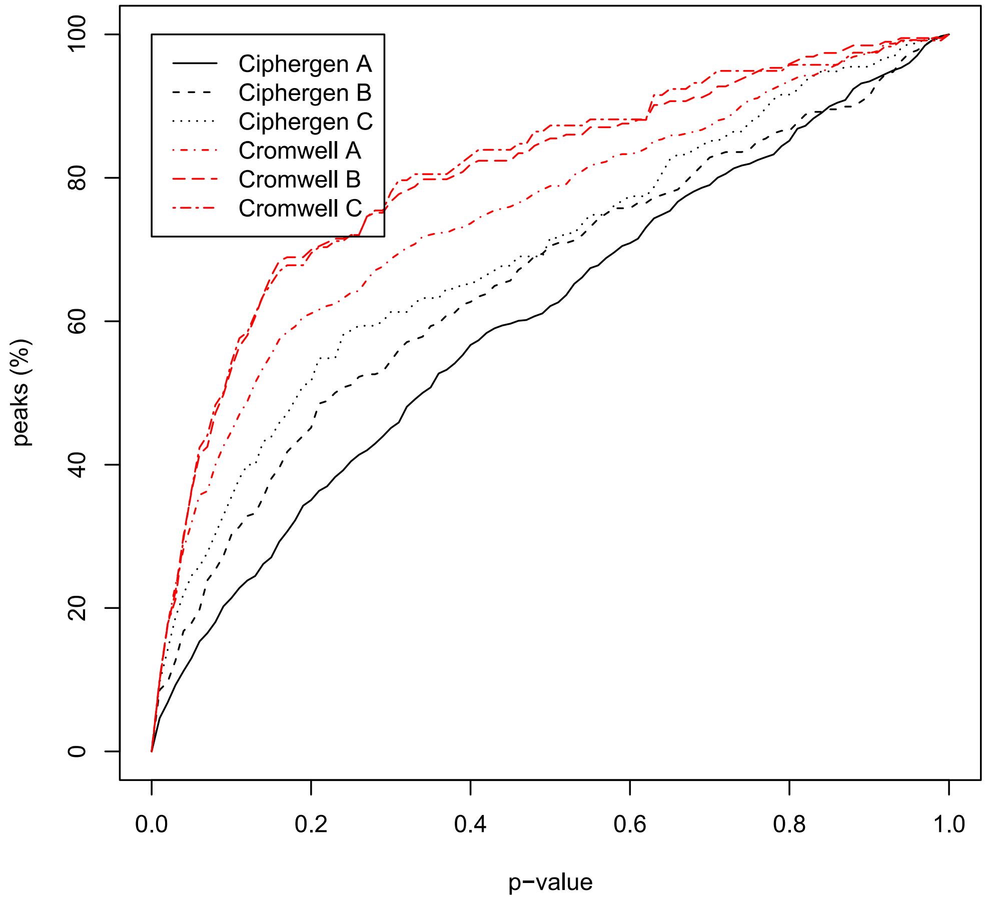

Cumulative plot of significance of detected peaks (ovarian cancer dataset. CM10). For each combination of pre-processing method and peak selection settings, the cumulative percentage of peaks with a p-value smaller than the value on the x-axis are shown. P-value of a peak is based on a t-test between the normalized intensities of the cancer and the control group.
