# Supplementary material for: Classification-based comparison of pre-processing methods for interpretation of mass spectrometry generated clinical datasets
Source: Proteome Sci. 2009 May 14;7:19. doi: 10.1186/1477-5956-7-19 (PMC2689848; doi:10.1186/1477-5956-7-19)
Supplement: Additional file 8 — Comparison of classifiers and pre-processing methods (ovarian cancer dataset). Each combination of chip type, pre-processing method and peak selection was ranked by its average classification accuracy on 1,000 test sets (size of training sets: 14, size of test sets: 14) for each classifier. The heatmap gives a colour coding of the ranks from 1 (highest accuracy, red) to 18 (lowest accuracy, light yellow). Columns of the heatmap are ranked by their average rank over all classifiers, with Ciphergen pre-processing using setting C and the combined CM10/Q10 data getting the highest rank. Classifiers are ordered by their average rank over all pre-processing combinations, with DLDA being the best ranked classifier. [file 1477-5956-7-19-S8.pdf]

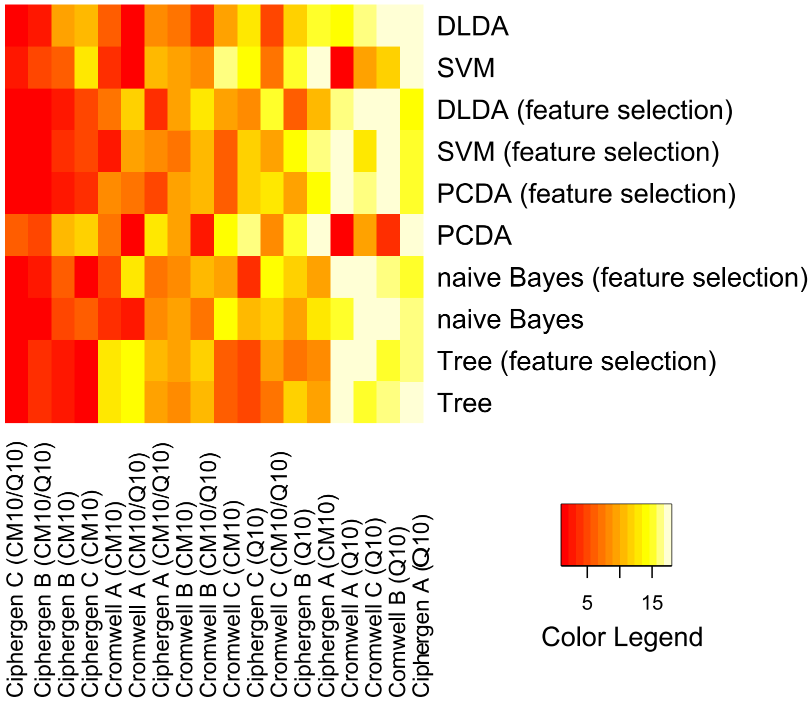

Comparison of classifiers and pre-processing methods (ovarian cancer dataset). Each combination of chip type, pre-processing method and peak selection was ranked by its average classification accuracy on 1,000 test sets (size of training sets: 14, size of test sets: 14) for each classifier. The heatmap gives a colour coding of the ranks from 1 (highest accuracy, red) to 18 (lowest accuracy, light yellow). Columns of the heatmap are ranked by their average rank over all classifiers, with Ciphergen pre-processing using setting C and the combined CM10/Q10 data getting the highest rank. Classifiers are ordered by their average rank over all pre-processing combinations, with DLDA being the best ranked classifier.
