# Supplementary material for: Classification-based comparison of pre-processing methods for interpretation of mass spectrometry generated clinical datasets
Source: Proteome Sci. 2009 May 14;7:19. doi: 10.1186/1477-5956-7-19 (PMC2689848; doi:10.1186/1477-5956-7-19)
Supplement: Additional file 9 — Comparison of classifiers and pre-processing methods (Gaucher dataset). Each combination of pre-processing method and peak selection was ranked by its average classification accuracy on 500 test sets (size of training sets: 27, size of test sets: 12) for each classifier. The heatmap gives a colour coding of the ranks from 1 (highest accuracy, red) to 6 (lowest accuracy, light yellow). Columns of the heatmap are ranked by their average rank over all classifiers, with Ciphergen pre-processing using setting A getting the highest rank. Classifiers are ordered by their average rank over all pre-processing combinations, with SVM being the best ranked classifier. [file 1477-5956-7-19-S9.pdf]

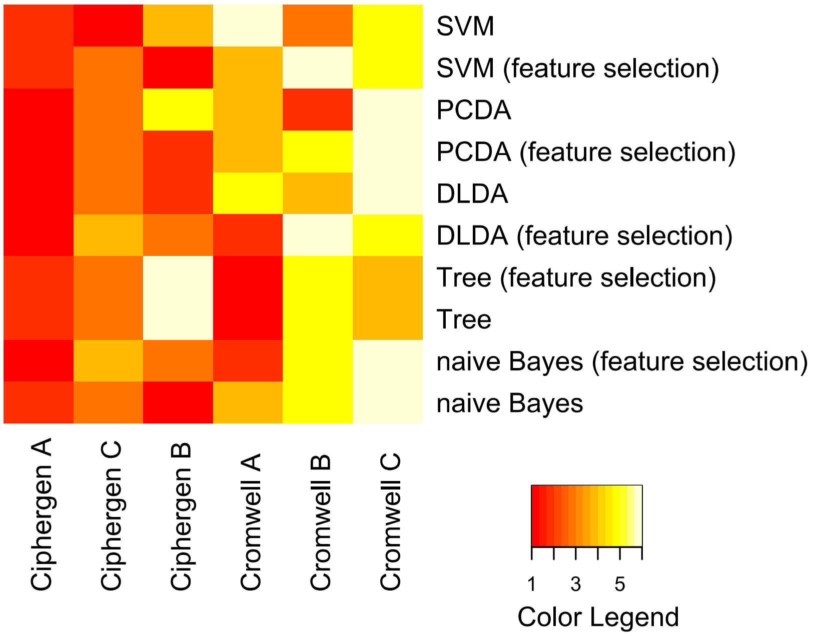

Comparison of classifiers and pre-processing methods (Gaucher dataset). Each combination of pre-processing method and peak selection was ranked by its average classification accuracy on 500 test sets (size of training sets: 27, size of test sets: 12) for each classifier. The heatmap gives a colour coding of the ranks from 1 (highest accuracy, red) to 6 (lowest accuracy, light yellow). Columns of the heatmap are ranked by their average rank over all classifiers, with Ciphergen pre-processing using setting A getting the highest rank. Classifiers are ordered by their average rank over all pre-processing combinations, with SVM being the best ranked classifier.
